# P-1225. The population Pharmacodynamics study of Ganciclovir in Thai kidney transplant recipients with cytomegalovirus infection

**DOI:** 10.1093/ofid/ofae631.1407

**Published:** 2025-01-29

**Authors:** Prawat Chantharit, Napun Sutharattanapong, Wasin Chatupho

**Affiliations:** Faculty of Medicine, Ramathibodi Hospital, Mahidol University and Keio University School of Medicine, Shinjuku-ku, Tokyo, Japan; Ramathibodi Hospital, Thailang, Krung Thep, Thailand; Ramathibodi Hospital, Thailang, Krung Thep, Thailand

## Abstract

**Background:**

The aim of this study was to describe the population pharmacodynamic (PPD) of intravenous Ganciclovir for preemptive and treatment in Thai kidney transplant recipients (KT) with cytomegalovirus (CMV) infection who were not during hemodialysis or KT-related systemic lupus erythematous.

Simulations of CMV viral load overtime according to recommended dose

**Figure:**
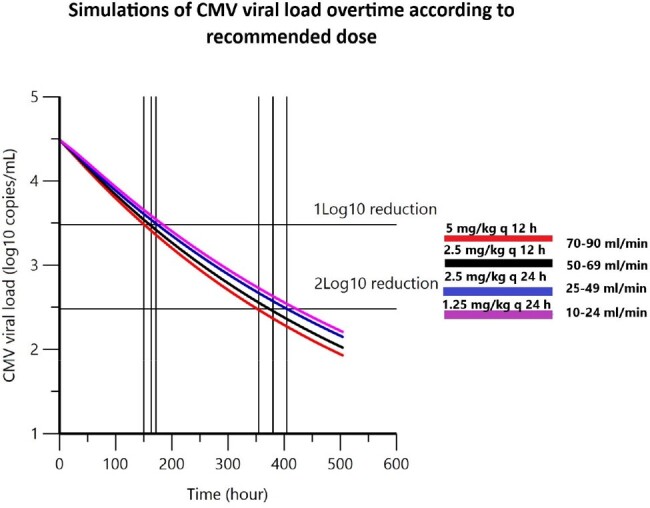

**Methods:**

A retrospective study was conducted by collecting data from medical records, from 1 January 2020-31 December 2022. The estimated pharmacokinetic (PK) parameters derived from published model were applied to link with PPD, subsequently simulate the changes of blood CMV viral load overtime according to recommended dosage, stratified by glomerular filtration rate (GFR). PK/PPD modelling, and simulation were performed with parametric methodology using Phoenix® software, version 8.4, Certara.

Visual Predictive Check

**Figure:**
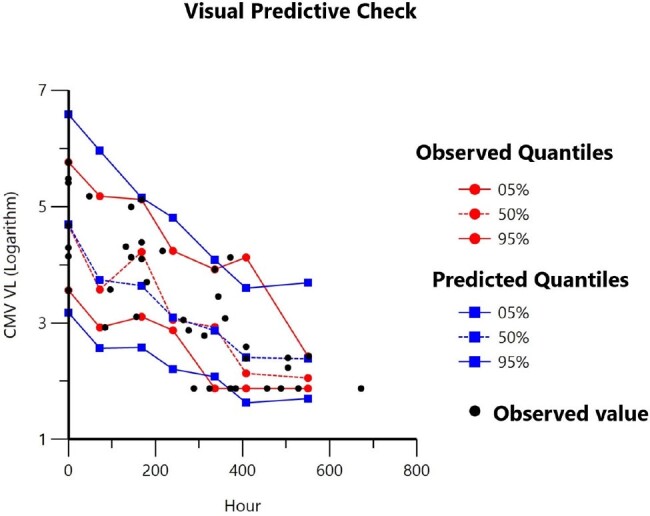

**Results:**

Twelve patients were included. The PPD model was developed by mechanistic indirect response equation, applying E_max_ model to stimulate the rate of CMV clearance. There were not any significant clinical and laboratory covariates affecting CMV dynamic. Goodness-of-fit plots were acceptable. A visual predictive check showed good consistency with observed data, whereas 1000 times-bootstrapping analysis was completed with 100% success rate. Ten thousand times-simulation indicated the time required for achieving 1-log_10_ and 2-log_10_ reduction of CMV viral load by recommended dose were 7-8 days and 15.8-18.5 days respectively. Viral decline was delayed in patients with lowered GFR.

Goodness-of-fit plot

**Figure:**
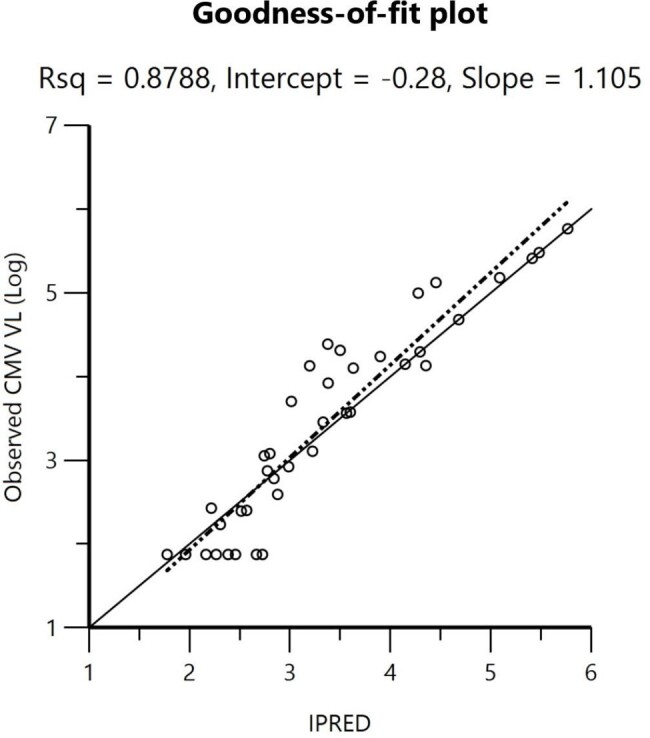

**Conclusion:**

The developed model described Ganciclovir-CMV viral load relationship and simulation result indicating the longer time for CMV viral load reduction in patients who had lower GFR. Our analysis demonstrated that the current recommended dose is required to be reconsidered and further systematic PD study is needed.

**Disclosures:**

**All Authors**: No reported disclosures

